# Role of ocular cytology in vernal keratoconjunctivitis

**DOI:** 10.1002/iid3.278

**Published:** 2019-12-05

**Authors:** Gaia Bruschi, Daniele G. Ghiglioni, Silvia Osnaghi, Chiara Rosazza, Denise Pires Marafon, Massimo Landi, Paola G. Marchisio

**Affiliations:** ^1^ Fondazione IRCCS Ca’ Granda Ospedale Maggiore Policlinico University of Milan Milan Italy; ^2^ Pediatric Highly Intensive Care Unit Fondazione IRCCS Ca’ Granda Ospedale Maggiore Policlinico Milan Italy; ^3^ Department of Ophthalmology Fondazione IRCCS Ca’ Granda Ospedale Maggiore Policlinico Milan Italy; ^4^ Paediatric Intermediate Care Unit Fondazione IRCCS Ca’ Granda Ospedale Maggiore Policlinico Milan Italy; ^5^ Pediatric National Healthcare System Turin – Istituto di Biomedicina e Immunologia molecolare Italian National Research Council Palermo Italy; ^6^ Department of Pathophysiology and Transplantation University of Milan Milan Italy

**Keywords:** eosinophils, ocular allergy, ocular cytology, pediatrics, vernal keratoconjunctivitis

## Abstract

**Background:**

Children with vernal keratoconjunctivitis (VKC) present symptoms that are similar to other ocular allergies, but more pronounced, and are controlled using topical steroids. To avoid excessive and prolonged use of topical steroid eye drops, over the past 20 years galenic eye drops of cyclosporine with a concentration of 1% to 2% and tacrolimus with a concentration of 0.1% have been introduced as a treatment for the severe and unresponsive forms. The main symptoms of VKC occur most frequently during the spring and tend to get worse during the summer, meaning that affected children tend to avoid exposure to sunlight. The aim of this study was to assess the most common cell types present in the conjunctiva of children with VKC, how ocular treatment can influence them, and whether affected children express a typical conjunctival pattern, which could be useful as a pathognomonic pattern of VKC, allowing us to study this rare eye disease.

**Method:**

This was a cohort study of 56 children, of whom 17 were not receiving any treatment at the time of testing, 14 were using steroid eye drops or had taken them in the previous 10 days, and 25 were treated with cyclosporine eye drops or tacrolimus eye drops 0.1%.

**Result:**

Children in group 1 (no topical therapy) express more epithelial cells, neutrophils, mast cells, eosinophils, and lymphocytes than the other two groups.

**Conclusion:**

Given the ease of performance, when conducting further longitudinal studies, the conjunctival cytology examination could be used, on the one hand, to diagnose VKC, especially when the clinical diagnosis is uncertain, and, on the other, to follow disease evolution and monitor the response to topical treatment.

## 1 INTRODUCTION

Vernal keratoconjunctivitis (VKC) is a chronic, bilateral, and asymmetric form of keratoconjunctivitis that primarily affects boys in the first decade of life.^1^, ^2^ The prevalence of VKC varies greatly and increases with vicinity to the equator. In Scandinavian countries, prevalence is close to 0%, whereas it reaches 0.27% in Italy^3^ and is higher still in Africa, with a reported prevalence of 6% in Ethiopia.^4^ Although the causes of VKC are still unclear, there is a strong correlation with exposure to sunlight, which exacerbates the disease.^5^

VKC is characterized by itch, photophobia, white mucous discharge, lacrimation, and foreign body sensations.^6^ There are three forms of VKC tarsal, limbal, and mixed. The tarsal form is characterized by the presence of papillae in the upper tarsal lid, and the limbal form by gelatinous infiltrate in the limbus and Horner‐Trantas dots.^4^

Although VKC usually resolves after puberty, it can lead to severe visual impairments if it is not adequately treated. Sight loss is reported in 5% to 30% of cases due to complications such as shield ulcers, corneal neovascularization, and keratoconus, and prolonged use of steroid therapy can cause cataracts and glaucoma.^7^

Normal conjunctival cytology is characterized by (a) epithelial cells, which are round or columnar; (b) sporadic neutrophils or lymphocytes; (c) absence of eosinophils and mast cells. In the event of inflammation, immune system cells reach the damage site through the vessels, disperse by diapedesis and gradually proliferate. Analyzing conjunctival scrapings can direct diagnosis towards an eye infection (eg, the presence of monocytes and/or macrophages indicates the possible presence of chlamydia, whereas lymphocytes may suggest a viral infection), tear film abnormalities (keratinized cells), or ocular allergy (eosinophils and mast cells). In VKC, conjunctival scrapings are characterized by the presence of eosinophils and mast cells, as in other ocular allergies, and few neutrophils.^8^

Small, intensely colored eosinophils have recently migrated to the inflammation site, whereas larger, less brightly colored eosinophils indicate degranulation.^9^, ^10^ The higher the number of inflammatory cells, the worse the ocular symptoms.^11^ Although the presence of eosinophils confirms the presence of an ocular allergy or VKC, their absence cannot exclude them.^12^

## 2 MATERIALS AND METHODS

This was an observational study on 56 children attending Fondazione IRCCS Ca’ Granda Ospedale Maggiore Policlinico di Milano for active VKC between June and September 2017. All of these patients were screened by conjunctival cytology. Diagnosis of VKC was performed by a pediatric ophthalmologist and the children were classified using the Bonini grading system.^6^

Each child was tested for (a) total serum immunoglobulin E (IgE); (b) presence of specific IgE for common allergens (birch, hazel, olive tree, grass, pellitory, ragweed, wormwood, *Dermatophagoides farinae*, *Dermatophagoides pteronyssinus*, *Alternaria*, cat and dog's dander), with a positive result >0.10 KUA/L; and (c) prick test for common inhalant allergens (birch, hazel, olive tree, grass, pellitory, ragweed, wormwood, *D. farinae*, *D. pteronyssinus*, *Alternaria*, cat and dog's dander).

The study was approved by the local Institutional Ethics Committee (Comitato Etico Milano Area 2) and was conducted in compliance with the ethical principles of the Declaration of Helsinki (no 312_2019). All the patients’ legal guardians gave their informed consent.

There were three treatment groups:

1) Group 1: 17 children who were not using immunomodulatory therapy or who had not used steroids for at least 10 days;
2) Group 2: 14 children treated with steroid eye drops;
3) Group 3: 25 children treated with cyclosporine 1% or tacrolimus 0.1% eye drops.

The following data were recorded for each patient: age, gender, atopy, inhalant‐specific IgE levels, or positive skin prick test.

Ocular cytology consists of (a) sampling; (b) fixation and staining; (c) microscopic exam. For sampling, we used a nasal curette (Rhino‐Probe). Sampling was performed on the posterior surface of the upper eyelid. We decided to sample the upper eyelid conjunctiva, rather than the lower eyelid, because the presence of papillae in this latter site is extremely rare, whereas they are almost always present on the upper lid.^13^ The curette was then scraped over a slide, that was fixed and stained with May‐Grunwald‐Giemsa and examined using an optical microscope. Each sample slide was examined for the presence of epithelial cells, eosinophils, neutrophils, mast cells, lymphocytes, bacteria, and fungi.

Each parameter was scored from 0 to 4, according to the Gelardi scoring system^14^

0: absence
½ + : occasional
1 + : few scattered cells, small clumps
2 + : moderate number, large clumps
3 + : large clumps not covering the field
4 + : clumps covering the entire field.

The results are presented as medians, quartiles (Q1 and Q3), and ranges. The differences between the groups were initially investigated with univariate analyses using Wilcoxon rank tests for differences in median values and *χ* ^2^ and Fischer exact tests for differences in frequency.

## 3 RESULTS

The age range was 5 to 15 years, with a median age of 9.4 years. Of the total, 71.4% of children were male, 78.1% had a mixed form of VKC, 18.1% a tarsal form, and 3.6% a limbal form. No patient had corneal involvement (shield ulcers or punctate keratitis) during the sampling period.

According to the Bonini grading system, 14.5% of children had mild‐intermittent VKC (grade 1, occasional use of antiallergy eye drops), 14.5% moderate, intermittent or persistent VKC (grade 2, daily use of antiallergy eye drops), 52.7% severe VKC (grade 3, not only daily use of antiallergy eye drops, but also of cyclosporine eye drops and topical pulsed steroids), 18.1% very severe VKC (grade 4, daily use of antiallergy eye drops, cyclosporine eye drops and topical high‐dose pulsed steroid eye drops to control the disease).^6^

Children in group 1 made only occasional use of antihistamine eye drops, mast cell stabilizers or dual‐acting agents. The most frequent product used in this group was ketotifen ophthalmic eye drops (90%).

Children in group 2 were using steroid eye drops at the time of sampling (dexamethasone sodium phosphate 0.15% eye drops in all cases).

Children in group 3 were using cyclosporine 1% eye drops two to three times a day (60%, 15 patients) or tacrolimus 0.1% eye drops twice a day (40%, 10 patients).

Seventy‐one percent had a parent with allergic rhinitis or asthma; 53% had positive prick tests for at least one allergen amongst birch, hazel, olive trees, grass, pellitory, ragweed, wormwood, *D. farinae*, *D. pteronyssinus*, *Alternaria*, and cat and dog dander, whereas 60% had specific IgE positivity for at least one of these allergens.

No significant differences were observed between the three treatment groups in terms of age, gender, positive family history of allergy, positive prick test, or specific IgE.

Conjunctival brushing cytology was performed for epithelial cells, neutrophils, eosinophils, and eosinophil degranulation, mast cells and mast cell degranulation, and lymphocytes. Each parameter was allocated a score of between 0 (absence) and 3 (strong presence).

Epithelial cells were more prevalent in group 1 (no topical therapy) than groups 2 and 3, with mean values of 3 (2‐3) in group 1, 2 (1‐2) in group 2, and 2 (1.5‐3) in group 3 (*P* values .0014 between groups 1 and 2, and .03 between groups 1 and 3).

Neutrophils were more prevalent in groups 1 and 3 than in group 2 (steroid therapy on‐going or used in the previous 10 days), with mean values of 1 (1‐1) in group 1, 1 (0.3‐1) in group 3, and 0.3 (0‐0.5) in group 2 (*P* values .0019 between groups 1 and 2, and .03 between groups 2 and 3).

Mast cells were more prevalent in group 1 than in groups 2 and 3, with mean values of 1 (1‐2), 1 (0‐1), and 1 (0.8‐2), respectively (*P* values .01 between groups 1 and 2, and .045 between groups 1 and 3).

There was no statistically significant difference in terms of mast cell degranulation in the three groups, with a mean score of 1 in all groups (*P* values .16 between groups 1 and 2, .43 between groups 2 and 3, and .45 between groups 1 and 3).

Eosinophils were less prevalent in the children in group 2 (0.3 [0‐1]) (steroid therapy on‐going or in the previous 10 days) and group 3 (0.5 [0‐1]) (cyclosporine or tacrolimus eye drops) than in group 1 (1 [0.5‐2]), with a statistically significant difference between groups 1 and 3 (*P* value .018).

As for mast cell degranulation, eosinophil degranulation did not show any statically significant difference between the groups, with scores of 0 (0‐0) in all three.

Lymphocytes were more prevalent in group 1, but without any statistically significant difference between the three groups (mean values of 1 [0‐1], 0 [0‐1], and 0 [0‐1] for groups 1, 2, and 3, respectively. *P* values .28 between groups 1 and 2, .10 between groups 1 and 3, .67 between groups 2 and 3).

We also calculated a “worse eye score,” that is, based on the neutrophil, mast cell, eosinophil, and lymphocyte count. The mean score was 4.5 (2.8‐5.3), 2.8 (0.5‐3.5), and 3.0 (1.8‐4.0) for groups 1, 2, and 3, respectively, with *P* values of .0015 between groups 1 and 2 and .03 between groups 1 and 3 (Table 1).

**Table 1:** Population characteristics

	Total (N = 56)	Group 1 (n = 17)	Group 2 (n = 14)	Group 3 (n = 25)	*P* value (1 vs 2)	*P* value (1 vs 3)	*P* value (2 vs 3)
Male	40 (71.4)	12 (70.6)	10 (71.4)	18 (72)	1.0	1.0	1.0
Age, y	9.4 (7.8‐11.8)	8.5 (7.7‐11.5)	8.6 (6.7‐11.1)	10.6 (8.5‐13.3)	.61	.09	.042
Family history of atopy	39/55 (70.9)	12/16 (75)	11 (78.6)	16 (64)	1.0	.51	.48
Serum IgE	196 (49‐547)	144 (38‐461)	166 (35‐479)	318 (118‐736)	.98	.13	.26
IgE >50 UI/mL	40/53	11/16 (68.8)	8/12 (66.7)	21 (84)	1.0	.44	.39
Positive prick test	29/55	9 (52.9)	6/13 (46.2)	14 (56)	1.0	1.0	.73
Positive RAST test	33/53	9/16 (56.3)	7/12 (58.3)	17 (68)	1.0	.52	.72
Worse eye							
Epithelial cells	2 (2‐3)	3 (2‐3)	2 (1‐2)	2 (1.5‐3)	.0014	.03	.16
Neutrophils	1 (0‐1)	1 (1‐1)	0.3 (0‐0.5)	1 (0.3‐1)	.0019	.19	.03
Mast cells	1 (0.6‐1)	1 (1‐2)	1 (0‐1)	1 (0.8‐2)	.01	.60	.045
Mast cell degranulation	1 (0‐2)	1 (0.5‐2)	1 (0‐1)	1 (0‐1.5)	.16	.43	.45
Eosinophils	0.5 (0‐1)	1 (0.5‐2)	0.3 (0‐1)	0.5 (0‐1)	.055	.018	.98
Eosinophil degranulation	0 (0‐0)	0 (0‐0)	0 (0‐0)	0 (0‐0)	.70	.60	.39
Lymphocytes	0 (0‐1)	1 (0‐1)	0 (0‐1)	0 (0‐1)	.28	.10	.67
Worse eye score	3.3 (1.5‐4.4)	4.5 (2.8‐5.3)	2.8 (0.5‐3.5)	3.0 (1.8‐4.0)	.0015	.03	.26

*Note*: Main findings at conjunctival scraping of the analyzed groups.

Abbreviation: IgE, Immunoglobulin E.

None of the analyzed conjunctival scrapings showed the presence of bacteria or fungi.

## 4 DISCUSSION

The normal cytological situation consists of conjunctival epithelial cells without cytoplasm or nuclear abnormalities. Epithelial cells have a roundish or columnar shape; flat cells originate from the conjunctiva. Inflammatory immune system cells are not usually found, except for a few sporadic neutrophils and lymphocytes. Eosinophils and mast cells are, therefore, absent both in the epithelium and in the tunica propria of the healthy conjunctiva, so the finding of these cells suggests a pathological process. In the presence of inflammation, a significant increase in inflammatory cells is caused by diapedesis from the conjunctival capillaries, with diverse and often characteristic representations of the various elements depending on the causes that gave rise to the pathological process. The number of inflammatory immune elements always correlates with the severity of the symptoms and the underlying conjunctival condition.

The diagnostic suspicion changed according to the prevailing cell type. Thus, whereas neutrophils are an indicator of infectious disease, such as bacterial or viral conjunctivitis, or an allergic or irritative condition, such as giant papillary conjunctivitis, lymphocytes are typical of adenoviral conjunctivitis, Herpes simplex virus (HSV), or drugs. Monocytes are frequently observed in conjunctivitis caused by *Chlamydia* or HSV, whereas macrophages and plasma cells are found in the presence of in chlamydial conjunctivitis. The presence of eosinophils and mast cells suggest allergic conjunctivitis or VKC.^11^, ^15^

Furthermore, the abundant presence of Th2 cytokines (interleukin‐4 [IL‐4] and IL‐5) and their receptors in tears and in the serum of VKC patients confirm the crucial role played by Th2‐type immune response in the onset and progression of the inflammation observed in VKC.^16^, ^17^, ^18^

IL‐2, interferon‐gamma, and tumor necrosis factor‐beta, cytokines secreted by Th1 cells, on the other hand, are not increased in VKC.^19^

Our study once again highlights that eosinophils could be used as a marker for ocular allergy.^15^ Indeed, in our cohort of children with VKC, like those published by Fanelli et al^11^ and Spadavecchia et al,^20^ ocular cytology shows an increase in eosinophils in the conjunctiva, whereas they are absent in healthy subjects. Moreover, our study clearly shows that appropriate topical therapy with steroid eye drops (children in group 2) or cyclosporine/tacrolimus eye drops underwent a statistically significant reduction in this cell type. Therefore, given the ease of performance, when conducting further longitudinal studies, conjunctival scraping and eosinophil tests could be used, on the one hand, to diagnose VKC (or other ocular allergies), especially when diagnosis is uncertain because it is based on clinical signs and symptoms alone, and, on the other hand, to follow the disease's evolution and monitor the efficacy of the ocular treatment.

As for eosinophils, children with VKC show an elevated presence of mast cells in the conjunctiva, especially those who are not receiving any eye treatment. When adequately treated, with either steroid eye drops (group 2) or cyclosporine or tacrolimus eye drops (group 3), this high concentration of mast cells showed a considerable decrease.

Our study did not reveal a statistically significant difference in terms of eosinophils and mast cell degranulation in the three groups studied.

Neutrophils were also far more prevalent in group 1 (no therapy children) than in the other groups (a fact that is particularly evident when group 1 [no therapy] is compared with group 2 [steroid eye drops], with a *P* value of .0019), which once again shows that immune‐mediated inflammation in VKC is a complex phenomenon that does not merely involve eosinophils and mast cells (ie, “allergic” cells), but also other immune system components. This could be why children with VKC show limited and partial response to antihistamine therapy alone, consisting in a reduction in reported itch only.

The presence of lymphocytes was higher than reported in previous studies on healthy subjects but without any statically significant differences between the three groups.

In our study, the evaluation of the “worse eye score” showed that appropriate ocular treatment is able to reduce the conjunctival cellularity of children with VKC, with both topical steroid therapy and cyclosporine or tacrolimus eye drops.

The presence or absence of prick test positivity, serum IgE elevation or common antigen‐specific IgE positivity did not influence the cellularity of ocular specimens, neither did a positive familiar history of rhinitis and asthma.

## 5 CONCLUSIONS

Like children with seasonal or perennial ocular allergies, those with VKC, present eosinophils and mast cells in the upper tarsal conjunctiva.

Children in group 1 (no topical therapy) express more epithelial cells, neutrophils, mast cells, eosinophils, and lymphocytes than the other two groups, showing that eye treatment can influence the composition of the conjunctiva in affected children.

Thus, given the ease of performance, when conducting further longitudinal studies, the conjunctival cytology examination and eosinophil test could be used, on the one hand, to diagnose VKC, especially when the clinical diagnosis is uncertain and, on the other hand, to follow disease evolution and monitor the response to topical treatment.

Confirmation of these results with large‐scale studies could not only lead to the development of personalized therapies but also provide greater insight into the pathogenesis of VKC.

## CONFLICT OF INTERESTS

The authors declare that there are no conflict of interests.
